# Investigating Silver Coordination to Mixed Chalcogen Ligands

**DOI:** 10.3390/molecules171113307

**Published:** 2012-11-08

**Authors:** Fergus R. Knight, Rebecca A.M. Randall, Lucy Wakefield, Alexandra M. Z. Slawin, J. Derek Woollins

**Affiliations:** School of Chemistry, University of St. Andrews, St Andrews, Fife KY16 9ST, UK

**Keywords:** silver(I) complexes, chalcogen-donor, coordination networks, helical chain polymer, acenaphthene ligands

## Abstract

Six silver(I) coordination complexes have been prepared and structurally characterised. Mixed chalcogen-donor acenaphthene ligands **L1**–**L3** [Acenap(EPh)(E'Ph)] (Acenap = acenaphthene-5,6-diyl; E/E' = S, Se, Te) were independently treated with silver(I) salts (AgBF_4_/AgOTf). In order to keep the number of variables to a minimum, all reactions were carried out using a 1:1 ratio of Ag/L and run in dichloromethane. The nature of the donor atoms, the coordinating ability of the respective counter-anion and the type of solvent used in recrystallisation, all affect the structural architecture of the final silver(I) complex, generating monomeric, silver(I) complexes {[AgBF_4_(L)_2_] (**1** L = **L1**; **2** L = **L2**; **3** L = **L3**), [AgOTf(L)_3_] (**4** L = **L1**; **5** L = **L3**), [AgBF_4_(L)_3_] (**2a** L = **L1**; **3a** L = **L3**)} and a 1D polymeric chain {[AgOTf(**L3**)]_n_ **6**}. The organic acenaphthene ligands **L1**-**L3** adopt a number of ligation modes (bis-monodentate μ_2_-η^2^-bridging, *quasi*-chelating combining monodentate and *η*^6^-E(phenyl)-Ag(I) and classical monodentate coordination) with the central silver atom at the centre of a tetrahedral or trigonal planar coordination geometry in each case. The importance of weak interactions in the formation of metal-organic structures is also highlighted by the number of short non-covalent contacts present within each complex.

## 1. Introduction

Coordination chemistry is an integral feature of inorganic and bioinorganic chemistry [1,2,3,4], with many applications in polymer design and materials science [4,5,6,7]. Following the pioneering work on transition metal chemistry by Nobel Prize winning Swiss chemist Alfred Werner, the metal-ligand interaction emerged as an important tool for the manufacture of supramolecular metal complexes and is prominent in the design of organic solids and metal-organic frameworks (MOFs) [4,5,6,7,8,9,10,11,12,13,14].

Crystal engineering utilises the metal-ligand coordination bond to construct coordination networks, generally through the self-assembly of tuneable building blocks [4,5,6,7,8,9,10,11,12,13,14]. Bridging organic ligands acting as rigid supports are linked in an ordered lattice, building extended and often multidimensional networks with central metal ions. Modification of the functional groups within the ligand shell can control the properties, topology and geometry of the extended network and lead to potential applications as new functional materials [10,11,12,13,14].

Nevertheless, the unpredictability of the polymeric architecture is a major challenge when designing supramolecular complexes. Self-assembly, which dictates the structural motif of the final complex is controlled by experimental conditions [10,11,12,13,14]. Factors such as the central metal ion oxidation state, the coordination geometry, the metal-to-ligand ratio, the nature and spacer length of the bridging ligand, the presence of solvents and the type of counter-anions, all play a significant role [10,11,12,13,14]. A subtle variation to any one of these parameters can influence the geometry of the final solid state structure, generating for example extended three-dimensional networks, linear chain polymers or simple monomeric species [10,11,12,13,14,15].

Silver has become a fashionable building block for connecting organic ligands in supramolecular networks [1,2,3,4,10,11,12,13,14]. The lack of stereochemical preference of a d^10^ configuration enables silver(I) to adopt a variety of coordination geometries (coordination number 2–8) and generate interesting supramolecular polymers with unique structural motifs [1,2,3,4,10,11,12,13,14,16]. The silver(I) cation primarily adopts either linear, trigonal or tetrahedral configurations and has the ability to form short Ag···Ag contacts; an important factor in the construction of metal organic frameworks [17].The availability of a number of silver(I) salts makes it easy to analyse the effects counter-anions have on the structure of supramolecular networks, such as their ability to coordinate to the metal centre [12,13,14].

**Figure 1:**
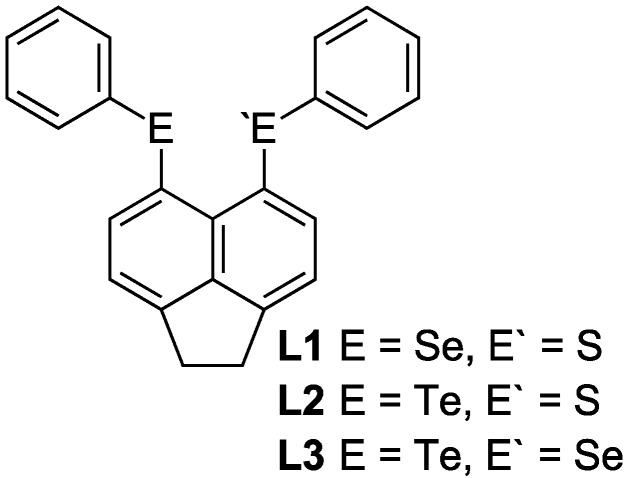
Acenaphthene chalcogen donor ligands Acenap[EPh][E'Ph] **L1**–**L3** [49,50,51].

Naphthalenes [18,19,20,21] and related 1,2-dihydroacenaphthylenes (acenaphthenes)[22] provide the perfect framework from which to design tunable donor ligands for thc preparation of metal complexes [23,24]. The rigidity of the organic backbone and the geometric constraints unique to these compounds, imposed by a double substitution at the close *peri*-positions, ensures metal coordination is favoured in order to achieve a relaxed geometry [24]. We have previously utilised the naphthalene backbone to prepare a variety of chalcogen and phosphorus compounds and associated metal complexes [25,26,27,28,29,30,31,32,33,34,35,36,37,38,39,40,41,42,43,44,45,46,47,48]. Our current work has focussed on the acenaphthene backbone, preparing halogen-chalcogen and chalcogen-chalcogen derivatives [49,50,51] and a series of halogen-tin compounds [52]. Coupled with silver(I) salts, the series of bromo-chalcogen compounds {[Acenap(Br)(EPh)] (E = S, Se, Te)} [49,50,51] were shown to form a variety of monomeric complexes [53],whilst the bis-chalcogen derivatives {[Acenap(EPh)] (E = S, Se)} [49,50,51] proved ideal building blocks for the assembly of supramolecular networks and extended structures, acting as bridging organic donor ligands between diversely coordinating silver(I) centres [54]. Herein we present a comparative structural study of the self-assembly of mixed chalcogen-donor ligands Acenap[EPh][E'Ph] (Acenap = acenaphthene-5,6-diyl; EE' = SeS, TeS, TeSe) **L1**–**L3** [49,50,51] (Figure 1) with silver tetrafluoroborate (AgBF_4_) and silver trifluoromethanesulfonate (AgOTf).

## 2. Results and Discussion

The three mixed acenaphthene derivatives Acenap[EPh][E'Ph] (Acenap = acenaphthene-5,6-diyl; EE' = **L1** SeS, **L2** TeS, **L3** TeSe [49,50,51], were each independently treated with silver tetrafluoroborate [AgBF_4_] and silver trifluoromethanesulfonate [AgOTf]. In order to keep the number of variables to a minimum, the reactions were carried out using a 1:1 ratio of Ag/**L** and run in dichloromethane under an oxygen- and moisture-free nitrogen atmosphere. The complexes **1**–**6** obtained were characterised by multinuclear NMR and IR spectroscopy and mass spectrometry and the homogeneity of the new compounds was where possible confirmed by microanalysis; ^77^Se and ^125^Te-NMR data can be found in Table 1. Crystal structures were determined for **1**–**6** and **2a** and **3a** (recrystallisation products of **2** and **3**, respectively). A number of the silver(I) complexes were found to be unstable towards light whilst in solution. Selected interatomic distances, angles and torsion angles are listed in Table 2 and Table 3. Hydrogen-bond and other non-conventioanl weak inter- and intra-moleular interaction data can be found in Table S1 in the Electronic Supporting Information (ESI). Further crystallographic information can be found in Table 4,Table 5,Table 6 and in Figures S1–S4 and Tables S2 and S3 in the ESI.

**Table 1:** ^77^Se and ^125^Te-NMR spectroscopic data ^[a]^.

	1	2	3	4	5	6
*Peri-atoms*	Se, S	Te, S	Te, Se	Se, S	Te, S	Te, Se
^77^Se-NMR	379.1	-	322.6	369.9	-	321.6
^125^Te-NMR	-	567.0	544.1	-	552.3	537.2
	**L1**	**L2**	**L3**			
^77^Se-NMR	433.7	-	340.7			
^125^Te-NMR	-	689.4	663.4			

^[a]^ Spectra of **2**, **5** and **6** run in CD_3_CN, spectra of **1** and **3** run in (CD_3_)_2_CO, spectrum of **4** run in CDCl_3_; δ (ppm).

**Table 2:** Selected interatomic distances [Å] and angles [°] for **1**–**6**.

Compound	1		2		3	4	5	6
Ligand; *peri*-atoms	L1; Se,S	L1; Se,S	L2; TeS	L2; TeS	L3; TeSe	L1; Se,S	L2; TeS	L3; TeSe
E···E'	3.1122(15)	3.1018(16)	3.1502(16)	3.1581(16)	3.2342(18)	3.14(4)	3.1669(10)	3.2417(11)
Σ*r*_vdW_—E···E' ^[a]^; % Σ*r*_vdW_^[a]^	0.588; 84	0.598; 84	0.710; 82	0.702; 82	0.726; 82	0.460; 87	0.693; 82	0.718; 82
*Peri-region bond angles*								
E(1)-C(1)-C(10)	122.1(4)	122.1(4)	122.4(4)	123.9(4)	124.9(7)	122(8)	122.6(2)	124.7(6)
C(1)-C(10)-C(9)	129.3(5)	129.3(5)	131.1(5)	129.0(5)	128.4(10)	130(12)	130.1(3)	130.2(7)
E'(1)-C(9)-C(10)	123.0(4)	122.8(5)	121.1(5)	121.4(4)	125.0(9)	124(12)	121.7(3)	121.6(6)
Σ of bay angles	374.4(11)	374.2(11)	374.6(11)	374.3(11)	378.3(20)	376(24)	374.4(6)	376.5(16)
Splay angle ^[b]^	14.4	14.2	14.6	14.3	18.3	16.0	14.4	16.5
*Out-of-plane displacement*								
E	−0.298(1)	−0.289(1)	−0.332(1)	−0.307(1)	−0.267(1)	0.375(1)	0.400(1)	0.173(1)
	0.177(1)	0.208(1)	0.150(1)	0.189(1)	0.143(1)	−0.183(1)	−0.114(1)	−0.393(1)
*Central naphthalene ring torsion angles*								
C:(6)-(5)-(10)-(1)	177.47(1)	177.67(1)	179.19(1)	177.11(1)	177.83(1)	179.27(1)	178.65(1)	178.77(1)
C:(4)-(5)-(10)-(9)	177.97(1)	178.20(1)	177.01(1)	178.65(1)	179.58(1)	175.92(1)	178.07(1)	176.60(1)

^[a]^ van der Waals radii used for calculations: *r*_vdW_(Br) 1.85Å, *r*_vdW_(S) 1.80Å, *r*_vdW_(Se) 1.90Å, *r*_vdW_(Te) 2.06Å [55]; ^[b]^ Splay angle: Σ of the three bay region angles—360.

**Table 3:** Selected silver coordination interatomic distances [Å] and angles [°] for **1**–**6**.

	1	2	3	4	5	6
Ag1-E1	2.6252(7)	2.7053(9)	2.7023(12)	2.628(14)	2.7304(8)	
Ag1-E1^1^				2.628(14)	2.7304(8)	
Ag1-E1^2^				2.628(14)	2.7304(8)	
Ag1-E2	2.6236(7)	2.7066(9)	2.7023(12)			
Ag1-cg(19-24)	3.002(1)	3.065(1)	3.119(1)			
Ag1-cg(49-54)	2.982(1)	3.054(1)	3.119(1)			
Ag1···Ag1^1^	8.481(1)	8.515(1)	8.856(1)	9.468(1)	9.728(1)	
E1-Ag1-E1^1^				119.5(4)	119.977(6)	
E1-Ag1-E1^2^				119.5(4)	119.977(6)	
E1^1^-Ag1-E1^2^				119.5(4)	119.977(6)	
E1-Ag1-E2	135.14(3)	140.40(3)	144.55(6)			
E1-Ag1-cg(19-24)	105.67(1)	104.67(1)	106.65(1)			
E1-Ag1-cg(49-54)	89.80(1)	88.57(1)	87.74(1)			
E2-Ag1-cg(19-24)	89.43(1)	88.13(1)	106.65(1)			
E2-Ag1-cg(49-54)	105.90(1)	104.83(1)	87.74(1)			
cg(19-24)-Ag1-cg(49-54)	139.32(1)	141.06(1)	132.09(1)			
Ag1-Te1						2.6807(11)
Ag1-Se1^1^						2.6013(12)
Ag1-O1						2.360(6)
Ag1···Ag1						5.929(1)
Te1-Ag1-Se1						132.70(4)
Te1-Ag1-O1						111.72(15)
Se1-Ag1-O1						112.75(15)

**Table 4:** Crystallographic data for **1**–**3**.

	1	2	3
Empirical Formula	C_48_H_36_AgBF_4_S_2_Se_2_·2CH_2_Cl_4_	C_48_H_36_AgBF_4_S_2_Te_2_·2CH_2_Cl_4_	C_48_H_36_AgBF_4_Se_2_Te_2_·CH_2_Cl_2_
Formula Weight	1199.39	1296.67	1305.54
Temperature (°C)	−180(1)	−180(1)	−180(1)
Crystal Colour, Habit	colourless, prism	colourless, platelet	colourless, platelet
Crystal Dimensions (mm^3^)	0.100 × 0.100 × 0.020	0.100 × 0.100 × 0.010	0.080 × 0.060 × 0.020
Crystal System	monoclinic	monoclinic	monoclinic
Lattice Parameters	a = 11.783(3) Å	a = 11.823(5) Å	a = 11.923(6) Å
	b = 28.585(5) Å	b = 29.132(9) Å	b = 29.68(2) Å
	c = 14.459(3) Å	c = 14.588(5) Å	c = 14.794(7) Å
	-	-	-
	β = 106.853(5)°	β = 107.227(9)°	β = 105.833(11)°
	-	-	-
Volume (Å^3^)	V = 4661(2)	V = 4799(3)	V = 5037(4)
Space Group	P2_1_/n	P21/n	C2/c
Z value	4	4	4
Dcalc (g/cm^3^)	1.709	1.795	1.721
F000	2384	2528	2504
μ(MoKα) (cm^−1^)	23.646	19.714	31.322
No. of Reflections Measured	28463	25411	15364
Rint	0.0409	0.0691	0.1069
Min and Max Transmissions	0.820–0.954	0.633–0.980	0.476–0.939
Observed Reflection (No. Variables)	8467(577)	8419(577)	4395(285)
Reflection/Parameter Ratio	14.67	14.59	15.42
Residuals: R_1_ (I > 2.00σ(I))	0.0521	0.0703	0.0786
Residuals: R (All reflections)	0.0712	0.1029	0.1527
Residuals: *w*R_2_ (All reflections)	0.1603	0.2006	0.2629
Goodness of Fit Indicator	1.081	1.083	1.029
Maximum peak in Final Diff. Map	1.93 e^−^/Å^3^	2.39 e^−^/Å^3^	1.21 e^−^/Å^3^
Minimum peak in Final Diff. Map	−0.98 e^−^/Å^3^	−1.82 e^−^/Å^3^	−0.75 e^−^/Å^3^

**Table 5:** Crystallographic data for **4**–**6**.

	4	5	6
Empirical Formula	C_73_H_54_AgF_3_O_3_S_4_Se_3_	C_73_H_54_AgF_3_OS_4_Te_3_	C_25_H_18_AgF_3_O_3_SSeTe·CH_2_Cl_2_
Formula Weight	1509.21	1623.13	854.83
Temperature (°C)	−180(1)	−180(1)	−180(1)
Crystal Colour, Habit	colourless, prism	yellow, prism	colourless, prism
Crystal Dimensions (mm^3^)	0.200 × 0.100 × 0.100	0.050 × 0.050 × 0.050	0.030 × 0.030 × 0.030
Crystal System	trigonal	trigonal	monoclinic
Lattice Parameters	a = 18.314(4) Å	a = 18.369(5) Å	a = 15.135(5) Å
	-	-	b = 10.141(3) Å
	c = 33.789(8) Å	c = 34.233(8) Å	c = 17.970(5) Å
	-	-	-
	-	-	β = 92.633(8)°
	-	-	-
Volume (Å^3^)	V = 9814(4)	V = 10004(5)	V = 2755(2)
Space Group	R-3	R-3	P2_1_/n
Z value	6	6	4
Dcalc (g/cm^3^)	1.532	1.616	2.061
F000	4536	4764	1640
μ (MoKα) (cm^−1^)	21.601	17.643	34.070
No. of Reflections Measured	20776	21511	17001
Rint	0.0522	0.0591	0.0700
Min and Max Transmissions	0.507–0.806	0.636–0.916	0.531–0.903
Observed Reflection (No. Variables)	3974(310)	4060(283)	5024(343)
Reflection/Parameter Ratio	12.82	14.35	14.65
Residuals: R_1_ (I > 2.00σ(I))	0.0797	0.0717	0.0535
Residuals: R (All reflections)	0.0965	0.0877	0.0678
Residuals: *w*R_2_ (All reflections)	0.2427	0.2092	0.1513
Goodness of Fit Indicator	1.061	1.200	1.102
Maximum peak in Final Diff. Map	1.88 e^−^/Å^3^	1.70 e^−^/Å^3^	1.49 e^−^/Å^3^
Minimum peak in Final Diff. Map	−0.84 e^−^/Å^3^	−1.70 e^−^/Å^3^	−1.39 e^−^/Å^3^

**Table 6:** Structural analysis of Ag(I) complexes **1**–**6**, **2a**, **3a** constructed from the self-assembly of [Acenap(EPh)(E'Ph)] **L1**–**L3** with AgBF_4_/AgOTf ^[a]^.

	Formula	Donor	Ag(I)	Ligand	Structural Architecture	
			Geometry	coordination		
**1**	[Ag(L1)_2_BF_4_]	SeS	Tetrahedral	*Quasi*-chelating;	Monomeric, mononuclear, 4-coordinate	
**2**	[Ag(L2)_2_BF_4_]	TeS			silver(I) sandwich complex containing a	
				monodentate,		
					bent metallocene type fragment and two	
**3**	[Ag(L3)_2_BF_4_]	TeSe		*η*6-E(phenyl)	*η*6- E(phenyl)-Ag interactions	
**4**	[Ag(L1)_3_CF_3_SO_3_]	SeS	Trigonal	Monodentate	Monomeric, mononuclear, 3-coordinate	
**5**	[Ag(L2)_3_CF_3_SO_3_]	TeS	planar		silver(I) complex	
**6**	[Ag(L3)CF_3_SO_3_]_n_	TeSe	Trigonal	Bis- monodentate	1D extended helical chain polymer	
			planar	μ_2_-η^2^-bridging,	containing one left-handed chain	
					[(-Ag-Te-C-C-C-Se-Ag-)n]	
**2a**	[Ag(L2)_3_BF_4_]	TeS	Trigonal	Monodentate	Monomeric, mononuclear, 3-coordinate	
**3a**	[Ag(L3)_3_BF_4_]	TeSe	planar		silver(I) complex	

^[a]^ In order to keep the number of variables to a minimum, the reactions were carried out using a 1:1 ratio of Ag/**L** and run in dichloromethane.

### 2.1. Reactions of Silver(I) Tetrafluoroborate

#### 2.1.1. [AgBF_4_(**L1**)_2_] **1**, [AgBF_4_(**L2**)_2_] **2** & [AgBF_4_(**L3**)_2_] **3**

**Figure 2:**
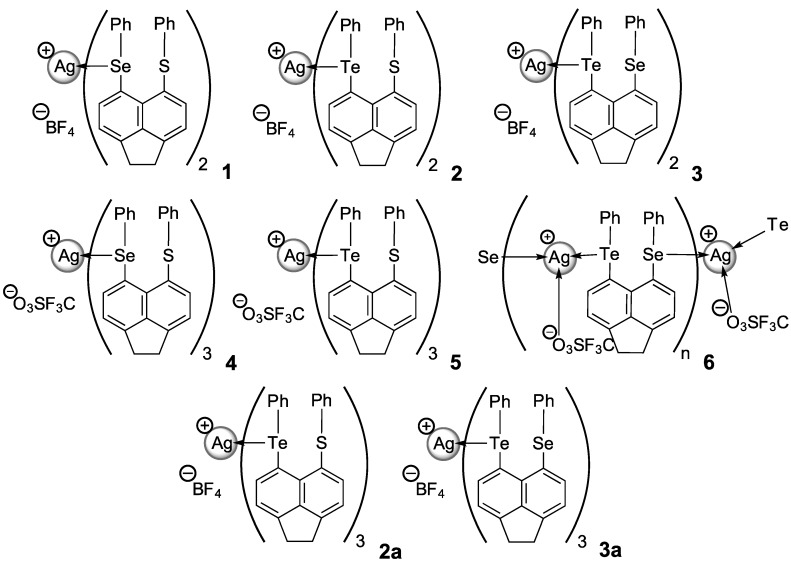
Silver(I) coordination complexes **1**–**6** (and **2a**, **3a** recrystallisation products of **2** and **3** respectively) prepared by the self-assembly of [Acenap(EPh)(E'Ph)] (E/E' = S, Se, Te) **L1**–**L3** with AgBF_4_ and AgOTf.

Treatment of mixed-chalcogen ligands [Acenap(SePh)(SPh)] **L1** and [Acenap(TePh)(SPh)] **L2** with one molar equivalent of AgBF_4_ afforded two isomorphous, two-coordinate, monomeric silver(I) complexes [Ag(BF_4_){Acenap(**L**)}_2_] (**1** (**L1**); **2** (**L2**); Figure 2). The corresponding reaction of [Acenap(TePh)(SePh)] **L3** with AgBF_4_ afforded a comparable two coordinate monomeric silver(I) complex [Ag(BF_4_){Acenap(**L3**)}_2_] **3**. Crystals suitable for X-ray diffraction were obtained in each case, by slow diffusion of hexane into a saturated dichloromethane solution of the respective product at room temperature in the absence of light. The two nearly identical asymmetric units of **1** and **2** contain four silver(I) centres, eight mixed-chalcogen ligands (**L1/L2**), four non-coordinating counter-anions (BF_4_^−^) and eight additional dichloromethane molecules. The asymmetric unit of **3** contains four silver(I) centres, eight **L3** ligands and four non-coordinating counter-anions (BF_4_^−^), but only four solvent molecules.

**Figure 3:**
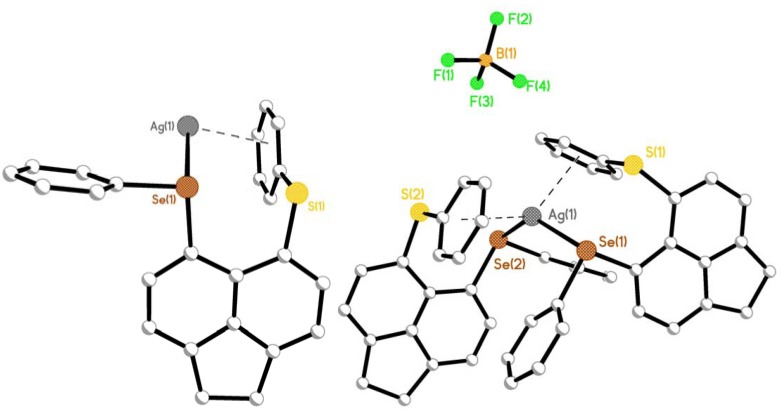
Two crystallographically distinct **L1** ligands bind to the silver(I) center via monodentate selenium coordination (left) to form complex **1** (right; H atoms and solvent molecules omitted for clarity). The structures of **2** and **3** (adopting similar conformations to **1**) are omitted here but can be found in Figure S1 in the ESI.

**Figure 4:**
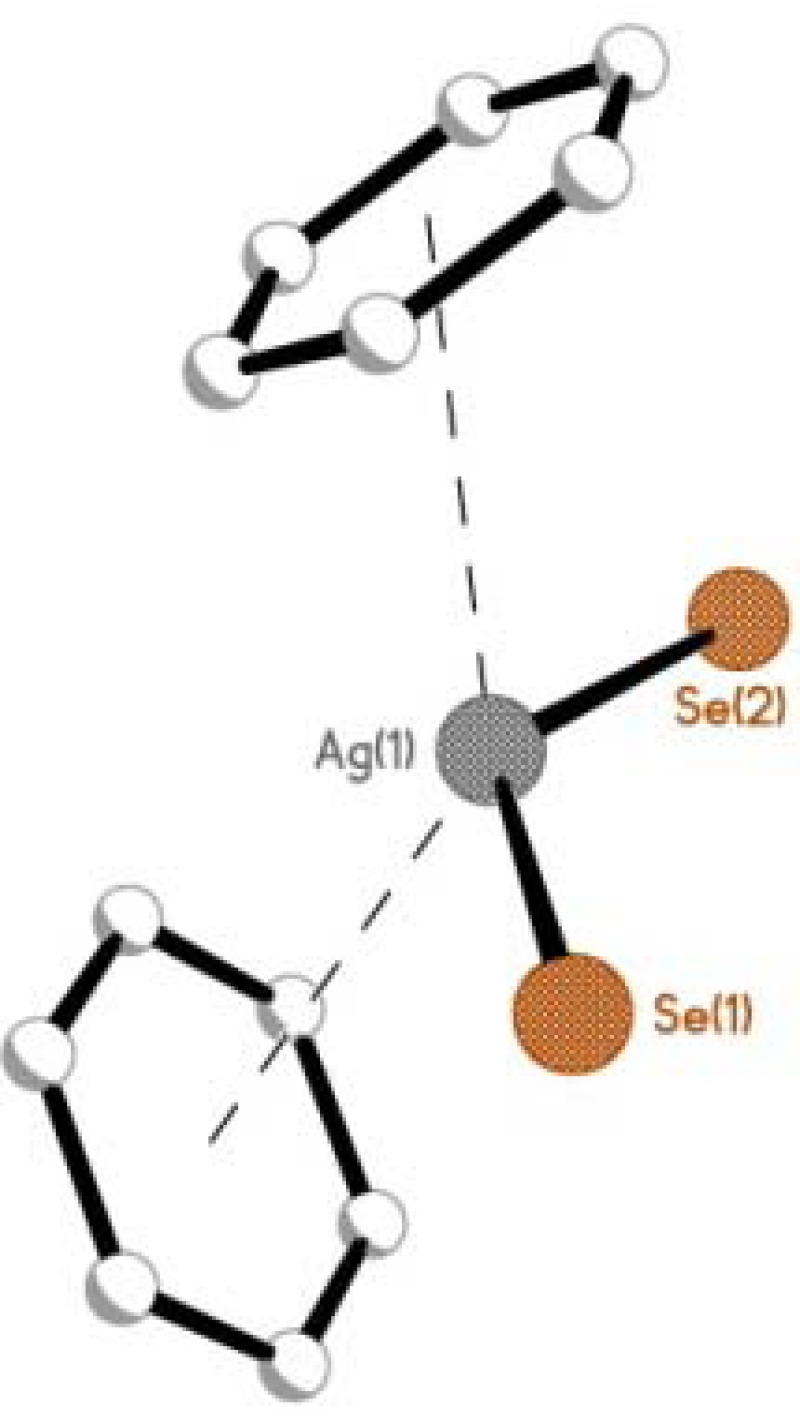
The bent metallocene motif found at the center of complex **1**, formed from two *η*^6^-S(phenyl)···Ag interactions. Comparative fragments found in complexes **2** and **3** are displayed in Figure S1, ESI.

Within the structural architecture of complexes **1** and **2**, two crystallographically unique molecules of the unsymmetrical mixed-chalcogen acenaphthene donor (**L1**/**L2**) act as monodentate ligands, binding in each case via the least electronegative chalcogen atom (Se/Te; Figure 3). The two-coordinate central silver atom adopts a distorted bent coordination geometry, with E(1)-Ag(1)-E(2) angles of 135.14(3)° **1** and 140.40(3)° **2**, respectively. An additional pair of weak *η*^6^-E(phenyl)···Ag interactions in the secondary coordination sphere completes a *quasi*-tetrahedral geometry around the central silver atom creating a bent metallocene type fragment within each sandwich complex [**1** Ag1-Se1 2.6252(7) Å, Ag1-Se2 2.6236(7) Å; Ag1···cg(19-24) 3.003(1) Å, Ag1···cg(49-54) 2.982(1) Å; angles in the range 89.4(1)–139.3(1)°; **2** Ag1-Te1 2.7053(9) Å, Ag1-Te2 2.7066(9) Å; Ag1···cg(19-24) 3.065(1) Å, Ag1···cg(49-54) 3.054(1) Å; angles in the range 88.1(1)–141.1(1)°; Figure 4]. Complex **3** adopts a comparable motif to **1** and **2** but as a consequence of the symmetry (crystallising in the monoclinic C2/c space group) only one crystallographically independent **L3** ligand is present within the crystal structure. [Ag1-Te1 2.7023(12) Å; Te1-Ag1-Te1^1^ 144.55(6)°; Ag1···cg(19-24) 3.119(1) Å; angles in the range 87.7(1)–155.4(1)°].

In all three structures the geometry around the silver centre is governed by the conformation of the rigid acenaphthene supports. The axial-equatorial conformation of the aromatic rings in both acenaphthene fragments of each complex (type AB) [56,57,58,59,60,61,62,63,64,65,66,67,68], positions the E-C_Ph_ bonds close to the acenaphthene plane with the secondary (E'-C_Ph_) bond aligned perpendicular to it [in each case χ(E) < χ(E'); E is the monodentate coordinating chalcogen donor]. The two facially bound axial E'(phenyl) rings are orientated parallel to their respective C_Acenap_-E'-C_Ph_ plane and subsequently linked to the adjacent silver centre via a *η*^6^-E'(phenyl)···silver type interaction to complete a *quasi*-chelate ring in each case (Figure 3). Coordination to silver has no significant effect on the conformation of the acenaphthene components or the degree of molecular distortion occurring within the organic frameworks of **1**–**3** compared with parent ligands **L1**–**L3** [49,50,51].The degree of distortion is related to the size of the atoms residing in the bay-region, with an expected lengthening of the *peri*-gap observed as the heavier congeners are located at the 5,6-positions along the series [**1** Se(1)···S(1) 3.1122(15) Å, Se(2)···S(2) 3.1018(16) Å (*cf.* 3.113(4) Å **L1**); **2** Te(1)···S(1) 3.1502(16) Å, Te(2)···S(2) 3.1581(16) Å (*cf.* 3.1576(15) Å **L2**); **3** Te(1)···Se(1) 3.2342(18) Å (*cf.* 3.2479(19) Å **L3**)] [49,50,51].

**Figure 5:**
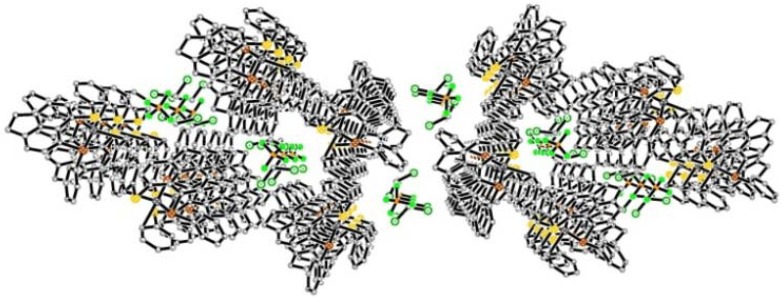
Complex **1** viewed down the z-axis; BF_4_^−^ counter-anions and dichloromethane solvent molecules stack in channels between the acenaphthene fragments. The packing of complexes **2** and **3**, viewed down the y-axis is displayed in Figure S, ESI2.

Neighbouring acenaphthene and phenyl rings in **1**–**3** stack along the z-axis and connect via weak CH···π interactions [2.73–2.95 Å] [69,70,71,72,73,74].Silver atoms subsequently align in columns, with the closest Ag···Ag distances between adjacent silver atoms of **1** 8.481(1) Å, **2** 8.515(1) Å, **3** 8.856(1) Å. The non-coordinating BF_4_^−^ counter-anions and dichloromethane solvent molecules interact via weak intermolecular CH···F interactions [2.27–2.52 Å], aligning to form weakly held 1D chains along the z-axis. The anion-solvent chains lie in the channels between the acenaphthene moieties and interact with the organic framework via additional CH···π, CH···F and CH···Cl interactions (Figure 5).

### 2.2. Reactions of Silver(I) Trifluoromethanesulfonate

#### 2.2.1. [AgOTf(**L1**)_3_] **4** & [AgOTf(**L2**)_3_] **5**

**Figure 6:**
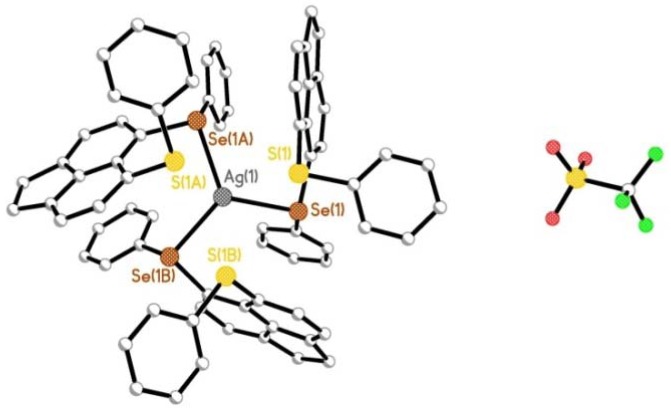
The three coordinate, mononuclear silver(I) complex **4** (H atoms omitted for clarity). The structure of **5** (adopting a similar conformation to **4**) is omitted here but can be found in Figure S3 in the ESI.

In contrast to the reactions with AgBF_4_, treatment of **L1** and **L2** with one molar equivalent of AgOTf afforded two isomorphous three-coordinate, monomeric, silver(I) complexes [Ag(OTf){Acenap(**L**)}_3_] (**4** (**L1**); **5** (**L2**); Figure 2 and Figure 6). Crystals suitable for X-ray diffraction were obtained by slow diffusion of hexane into a saturated dichloromethane (**4**), dichloromethane/methanol (**5**) solution of the respective product. Recrystallisations of both products were performed at room temperature, in the absence of light to prevent the complexes from decomposing. The two nearly identical asymmetric units contain six silver(I) centres, eighteen mixed-donor ligands (**L1/L2**) and interestingly six non-coordinating triflate counter-anions.

**Figure 7:**
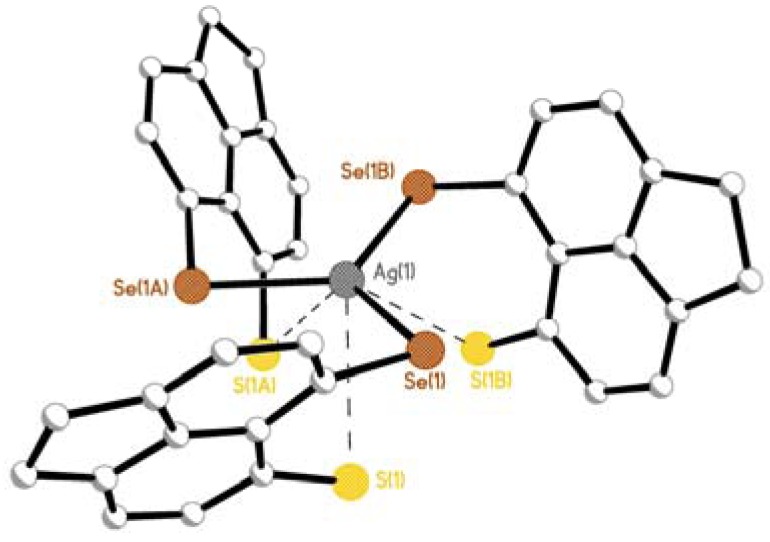
Weak Ag1···S1 contacts in the secondary coordination sphere affords a distorted *quasi*-trigonal prismatic geometry around the central silver atom in **4** and **5** **(**phenyl rings and H atoms omitted for clarity; complex **4** shown).

**Figure 8:**
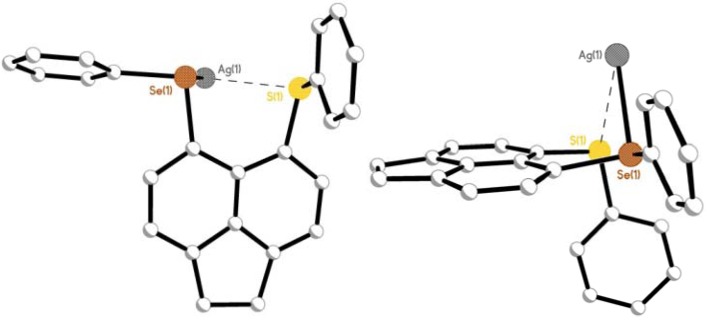
Short Ag1···S1 contacts in **4** and **5** construct 6-membered *quasi*-chelate rings which adopt twisted envelope type conformations (H atoms omitted for clarity; complex **4** shown).

Similar to **3**, complexes **4** and **5** crystallise in the monoclinic C2/c space group ensuring only one crystallographically independent ligand is present in each crystal structure; three crystallographically identical molecules of the respective ligand (**L1/L2**) thus bind with silver via monodentate Se/Te coordination to afford the monomeric complex. The central silver atom adopts a classical trigonal planar coordination geometry, lying 0.179(1) Å and 0.042(1) Å above the mean E1-E1^1^-E1^2^ plane in **4** and **5**, respectively [**4** Ag1-Se1 2.628(14) Å, Se1-Ag1-Se11 119.5(4)°; **5** Ag1-Te1 2.7304(8) Å, Te1-Ag1-Te11 119.977(6)°; Figure 6]. In the secondary coordination sphere, additional intramolecular Ag1···S contacts [**4** 3.42(3) Å; **5** 3.5986(10) Å], shorter than the sum of van der Waals radii for the two interacting atoms [55], complete a *quasi*-chelate ring with the central silver atom which assumes a distorted trigonal prismatic geometry [**4** Se1-Ag1-S1 angles 60.88(1)°, 89.07(1)°, 130.97(1)°, S1-Ag1-S11 70.26(1)°; **5** Te1-Ag1-S1 angles 58.13(1)°, 86.07(1)°, 129.08(1)°, S1-Ag1-S11 71.38(1)°; Figure 7 and Figure 8]. The AgESC_3_ six-membered *quasi*-chelate rings subsequently formed adopt twisted envelope type conformations, hinged about the E···S vectors. In each case, E1, C1, C9, C10 are essentially coplanar with S1 lying **4** 0.374(1) Å and **5** 0.317(1) Å below the plane and Ag1 sitting in the *peri*-gap, displaced **4** 2.378(1) Å and **5** 2.554(1) Å above the plane. The Ag1-E1-S1 plane in **4** is inclined by 97.53(1)°, with a more acute angle of 93.90(1)° displayed by **5** (Figure 8).

Within the structural architecture adopted by complexes **4** and **5**, each acenaphthene component adopts an axial-equatorial conformation of aromatic rings (type AB) [56,57,58,59,60,61,62,63,64,65,66,67,68], aligning the E-C_Ph_ bond along the organic backbone and the S-C_Ph_ bond perpendicular to the plane and directed away from the centre of the molecule. In both cases, coordination to silver has a limited impact on the degree of molecular distortion occurring in the organic backbone, with observed non-bonded intramolecular E1···S1 distances (**4** 3.14(4) Å; **5** 3.1669(10) Å) comparable to those of the free ligands **L1** (3.113(4) Å) and **L2** (3.1576(15) Å).[49,50,51] Within the extended structure, adjacent molecules interact via weak CH···π interactions [C3-H3···cg(19-24) **4** 2.62 Å, **5** 2.68 Å; C8-H8···cg(19-24) **4** 2.91 Å, **5** 2.90 Å] [69,70,71,72,73,74], stacking neighbouring silver centres in columns along the z-axis, with closest intermolecular Ag···Ag contacts **4** 9.468(1) Å and **5** 9.728(1) Å.

#### 2.2.2. [AgOTf(**L3**)]_n_ **6**

**Figure 9:**
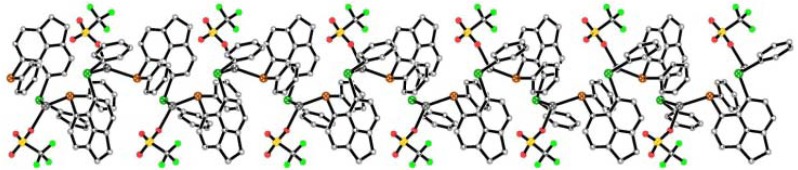
View of the 1D extended helical chain polymer **6** along the x-axis (H atoms and solvent molecules omitted for clarity).

In stark contrast to the previously described set of reactions, treatment of the mixed selenium-tellurium ligand [Acenap(TePh)(SePh)] **L3** with AgOTf afforded a mononuclear one-dimensional (1D) extended helical chain polymer [Ag(CF_3_SO_3_){Acenap(TePh)(SePh)}]*_n_* **6** (Figure 9). Crystals suitable for X-ray diffraction were obtained by slow diffusion of hexane into a saturated solution of **6** in dichloromethane, at room temperature in the absence of light. The asymmetric unit contains four silver(I) centres, four mixed tellurium-selenium **L3** ligands, four coordinating triflate counter-anions and four dichloromethane solvent molecules.

**Figure 10:**
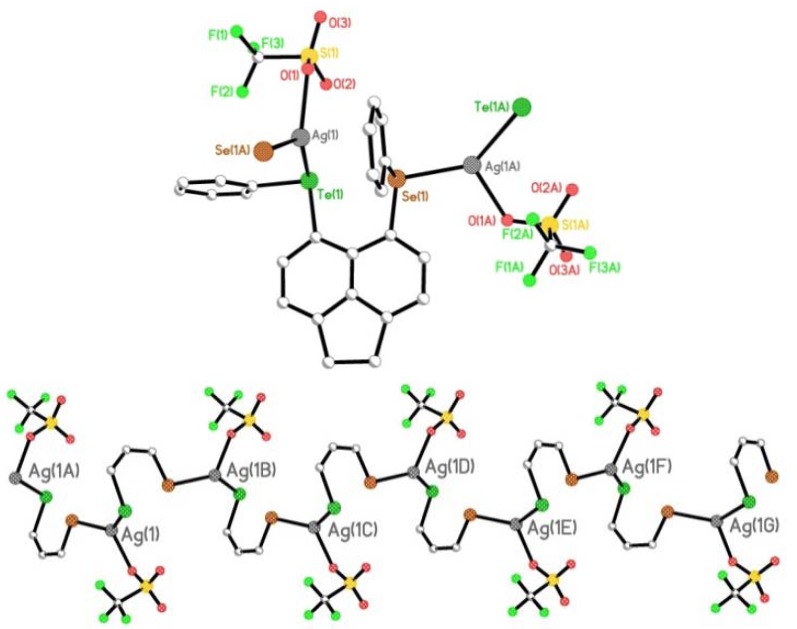
The repeating unit of extended helical chain polymer **6** (**top**; H atoms and solvent molecules omitted for clarity) and the central core of the repeating unit showing the three coordinate, trigonal planar silver(I) geometry (**bottom**).

Within the extended structure, the μ_2_-η^2^-bridging **L3** ligand binds simultaneously through both tellurium and selenium to independent silver(I) ions via bis-monodentate coordination (Figure 10). In turn, each silver(I) centre is coordinated to a Te and Se atom from two independent **L3** ligands, with the coordination of a single O atom of a neighbouring triflate molecule completing a *quasi*-trigonal planar geometry; Te1, Se1, O1, are coplanar with Ag1 displaced 0.246(1) Å above the mean Te1-Se1^1^-O1 plane [Ag1-Te1 2.6807(11) Å, Ag1-Se1^1^ 2.6013(12) Å, Ag1-O1 2.360(6) Å; X-Ag1-Y 111.72(15)°-132.70(4)°; Figure 10].

**Figure 11:**
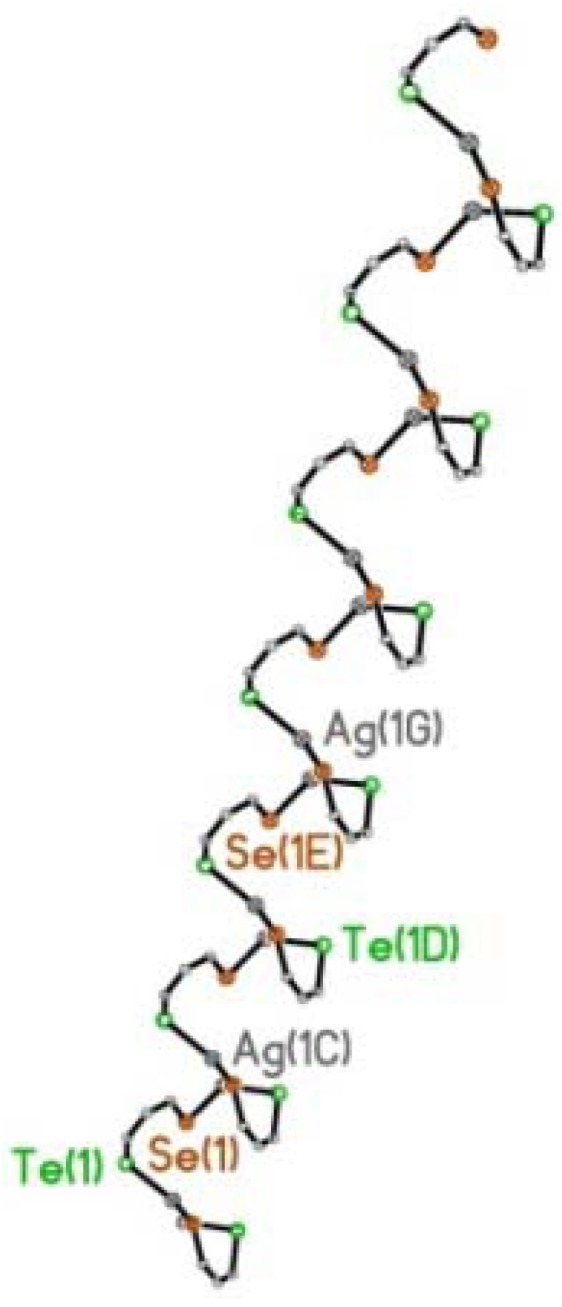
.Silver(I) ions interconnect via Te-C-C-C-Se bridges from independent **L3** ligands to form a left-handed helix [(-Ag-Te-C-C-C-Se-Ag-)*_n_*].

**Figure 12:**
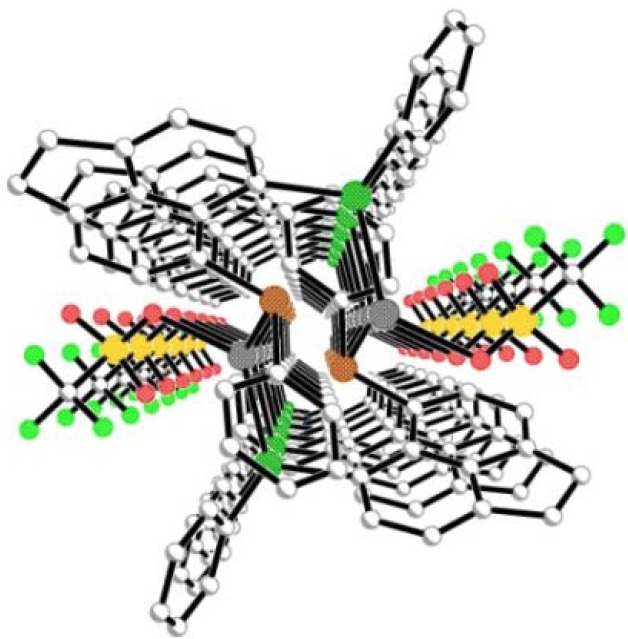
View of the 1D extended helical chain polymer **6** along the z-axis; silver atoms align in two columns with the closest non-bonding Ag···Ag distance 5.929(1) Å.

Repeating (**L3**AgOTf)*_n_* units assembled from μ_2_-η^2^-bridging **L3** ligands, propagate along the z-axis forming one-dimensional (1D) chains incorporating a single C-Te-Se-Ag helix (Figure 9,Figure 10,Figure 11). Silver(I) ions interconnect via Te-C-C-C-Se bridges from independent **L3** ligands to form a left-handed helix [(-Ag-Te-C-C-C-Se-Ag-)*_n_*; Figure 11]. The silver atoms align in two columns with the closest non-bonding Ag···Ag distance 5.929(1) Å (Figure 12). Within each helical chain weak non-bonding intermolecular CH···π interactions [69,70,71,72,73,74] exist between H14 and centroid cg(19–24) of neighbouring acenaphthene fragments [2.81 Å]. Two additional weak CH···O type interactions between acenaphthene and triflate moieties also participate in the construction of the helix [H8···O1 2.56 Å; H23···O2 2.57 Å].

Similarly, parallel helical chains aligning along the z-axis (Figure 12) also interact via weak CH···π interactions [69,70,71,72,73,74] between neighbouring acenaphthene fragments [C12-H12···cg(5-10) 2.92 Å], whilst CH···O interactions link acenaphthenes with triflate anions from the next helical chain [C11-H11···O1 2.40 Å]. Dichloromethane solvent molecules locate in channels formed by neighbouring helical chains and weakly coordinate to triflate anions via CH···O interactions [C26A-H26A···O3 2.68 Å].

Upon coordination to silver no significant alteration is observed to the acenaphthene backbone, with only a minor reduction in the non-bonding Te···Se distance from 3.2479(19) Å in the free ligand **L3** [49,50,51] to 3.2417(11) Å in **6**. The Te-C_Ph_ bond adopts an equatorial arrangement aligning along the mean plane with the Se-C_Ph_ bond occupying an axial orientation, perpendicular to the plane [68]. This configuration (type AB, comparable to **L3**) [56,57,58,59,60,61,62,63,64,65,66,67] aligns the Se(phenyl) ring close to the acenaphthene backbone promoting a close intramolecular CH···π interaction [H24···cg(5-10) 2.95 Å] [69,70,71,72,73,74].

#### 2.2.3. [AgBF_4_(**L2**)_3_] **2a** & [AgBF_4_(**L3**)_3_] **3a**

Experimental conditions such as central metal ion oxidation state, the metal-to-ligand ratio, the nature and spacer length of the bridging ligand, the presence of solvents and the type of counter-anions can have a profound influence on the structural architecture of the final complex and adds unpredictability to the self-assembly process [10,11,12,13,14]. Techniques and solvents used in the recrystallisation process can also affect the outcome of the final product. A subtle adjustment to the recrystallisation solvent systems for complexes **2** and **3** afforded two nearly identical three-coordinate, mononuclear, monomeric silver(I) complexes [Ag(BF_4_){Acenap(TePh)(EPh)}_3_] (**2a** E = S, **3a** E = Se) with structures analogous to complexes **4** and **5**. Crystals suitable for X-ray diffraction were obtained by slow diffusion of hexane into saturated dichloromethane/methanol (**2**) and tetrahydrofuran (**3**) solutions of the respective product. Further information on the crystal structures of **2a** and **3a** can be found in the ESI.

## 3. Experimental

### 3.1. General

All experiments were carried out under an oxygen- and moisture-free nitrogen atmosphere using standard Schlenk techniques and glassware. Reagents were obtained from commercial sources and used as received. Dry solvents were collected from a MBraun solvent system. Elemental analyses were performed by Stephen Boyer at the London Metropolitan University. Infra-red spectra were recorded as KBr discs in the range 4000–300 cm^−1^ on a Perkin-Elmer System 2000 Fourier transform spectrometer. ^1^H- and ^13^C-NMR spectra were recorded on a Jeol GSX 270 MHz spectrometer with δ(H) and δ(C) referenced to external tetramethylsilane. ^77^Se and ^125^Te-NMR spectra were recorded on a Jeol GSX 270 MHz spectrometer with δ(Se) and δ(Te) referenced to external Me_2_Se and Me_2_Te respectively, with a secondary reference for δ(Te) to diphenyl ditelluride [δ(Te) = 428 ppm]. ^19^F-NMR spectra were recorded on a Bruker Ultrashield 400 MHz spectrometer with δ(F) referenced to external trichlorofluoromethane. Assignments of ^13^C and ^1^H-NMR spectra were made with the help of H-H COSY and HSQC experiments. All measurements were performed at 25 °C. All values reported for NMR spectroscopy are in parts per million (ppm). Coupling constants (J) are given in Hertz (Hz). Mass spectrometry was performed by the University of St. Andrews Mass Spectrometry Service. Electrospray Mass Spectrometry (ESMS) was carried out on a Micromass LCT orthogonal accelerator time of flight mass spectrometer.

*[AgBF_4_{Acenap(SePh)(SPh)}_2_]* (**1**). To a solution of AgBF_4_ (0.10 g, 0.53 mmol) in dichloromethane (20 mL) was added [Acenap(SePh)(SPh)] (0.22 g, 0.53 mmol) in one batch at −30 °C. The reaction mixture was stirred at this temperature for 3 h and then at room temperature for a further 12 h. The solvent was removed *in vacuo*. The crude product was washed with diethyl ether and the brown precipitate which formed was collected by filtration. An analytically pure sample was obtained by recrystallization from diffusion of hexane into a saturated dichloromethane solution of the product (0.28 g, 97%); m.p. 200–202 °C (decomp); ^1^H-NMR [270 MHz, (CD_3_)_2_CO, 25 °C, TMS] *δ* = 7.84 (2 H, d, ^3^*J* (H,H) = 7.3, 2 × Acenap 4-H), 7.66–7.59 (4 H, 2 × Se*Ph* 12,16-H), 7.59–7.50 (2 H, m, 2 × Se*Ph* 14-H), 7.50–7.36 (6 H, m, 2 × Acenap 7-H, 2 × Se*Ph* 13,15-H), 7.35–7.25 (4 H, m, 2 × S*Ph* 19-21-H), 7.25–7.14 (4 H, m, 2 × Acenap 3-H, 2 × S*Ph* 20-H), 7.14–7.00 (6 H, m, 2 × Acenap 8-H, 2 × S*Ph* 18,22-H), 3.50–3.33 (8 H, m, 4 × C*H*_2_); ^13^C-NMR [67.9 MHz, (CD_3_)_2_CO, 25 °C, TMS]: *δ* = 149.5(q), 146.0(q), 143.3(q), 142.4(s), 140.9(q), 137.4(s), 133.5(s), 131.7(s), 131.4(s), 130.8(s), 130.2(q), 127.8(s), 127.5(s), 127.3(q), 122.5(s), 122.4(s), 118.5(q), 112.9(q), 31.4(s, *C*H_2_), 30.2(s, *C*H_2_); ^77^Se-NMR [51.5 MHz, (CD_3_)_2_CO, 25 °C, PhSeSePh]: *δ* = 379.1 (s); ^19^F-NMR [376.5 MHz, (CD_3_)_2_CO, 25 °C, CCl3F] −152.2(br s, ^10^BF_4_^−^) *δ* = − 152.3(br s, ^11^BF_4_^−^); IR (KBr disk): *v*_max_ cm^−1^: 2951w, 2930w, 2916w, 2840w, 2823w, 2376w, 2333w, 2118w, 2055w, 1636w, 1589s, 1574s, 1474s, 1436s, 1414s, 1325s, 1301s, 1282w, 1258w, 1234w, 1208w, 1157s, 1090vs, 1054vs, 838s, 816s, 745vs, 686vs, 625w, 606s, 552w, 519s, 459s, 401w, 351w, 309w; MS (ES^+^): *m/z* (%): 942.61 (100) [M^+^−BF_4_^−^]; elemental analysis calcd (%) for C_48_H_36_AgBF_4_S_2_Se_2_: C 55.9, H 3.5; Found: C 55.8, H 3.5.

*[AgBF_4_{Acenap(TePh)(SPh)}_2_]* (**2**). Complex **2** was obtained following the method described previously for **1** but with AgBF_4_ (0.12 g, 0.64 mmol), [Acenap(TePh)(SPh)] (0.30 g, 0.64 mmol). An analytically pure sample was obtained by recrystallization from diffusion of hexane into a saturated dichloromethane/methanol solution of the product (0.34 g, 94%); m.p. 125–127 °C (decomp); ^1^H-NMR [270 MHz, CD_3_CN, 25 °C, TMS] *δ* = 7.85 (1 H, d, ^3^*J* (H,H) = 7.2, Acenap 4-H), 7.53–7.46 (2 H, m, Te*Ph* 12,16-H), 7.44 (1 H, d, ^3^*J* (H,H) = 7.4, Acenap 3-H), 7.34 (1 H, d, ^3^*J* (H,H) = 7.2, Acenap 7-H), 7.32–7.12 (7 H, m, Acenap 8-H, Te*Ph* 13-15-H, S*Ph* 19-21-H ), 7.03–6.95 (2 H, m, S*Ph* 18,22-H), 3.35–3.22 (4 H, m, 2 × C*H*_2_); ^13^C-NMR [67.9 MHz, CD_3_CN, 25 °C, TMS]: *δ* = 151.6(q), 148.0(q), 144.0(q), 140.9(s), 139.6(s), 136.6(q), 134.9(q), 133.4(q), 132.7(q), 131.0(s), 129.7(s), 129.3(s), 129.1(s), 126.3(s), 126.1(s), 125.9(s), 121.4(s), 119.6(q), 29.7(s, *C*H_2_), 29.1(s, *C*H_2_); ^125^Te-NMR [81.2 MHz, CD_3_CN, 25 °C, PhTeTePh]: *δ* = 567.0(s); ^19^F-NMR [376.5 MHz, CD_3_CN, 25 °C, CCl_3_F]: *δ* = −152.3(br s, ^10^BF_4_^−^), -152.4(br s, ^11^BF_4_^−^); IR (KBr disk): *v*_max_ cm^−1^: 2989w, 2926w, 2828w, 2664w, 2531w, 2380w, 2266w, 1946s, 1881s, 1729w, 1631w, 1593s, 1572s, 1474s, 1434s, 1408w, 1333s, 1300w, 1272w, 1232w, 1210w, 1180w, 1155w, 1054vs, 996vs, 845s, 816w, 737vs, 689vs, 625w, 603w, 580w, 549w, 519w, 501w, 488w, 455w, 402w; MS (ES^+^): *m/z* (%): 1040.19 (100) [M^+^−BF_4_^−^]; elemental analysis calcd (%) for C_48_H_36_AgBF_4_S_2_Te_2_: C 51.0, H 3.2; Found: C 50.9, H 3.1.

*[AgBF_4_{Acenap(TePh)(SePh)}_2_]* (**3**). Complex **3** was obtained following the method described previously for **1** but with AgBF_4_ (0.12 g, 0.63 mmol), [Acenap(TePh)(SePh)] (0.32 g, 0.63 mmol). An analytically pure sample was obtained by recrystallization from diffusion of hexane into a saturated dichloromethane solution of the product (0.34 g, 88%); m.p. 100–105 °C (decomp); ^1^H-NMR [270 MHz, (CD_3_)_2_CO, 25 °C, TMS]: *δ* = 8.73 (1 H, d, ^3^*J* (H,H) = 7.6, Acenap 4-H), 8.15 (1 H, d, ^3^*J* (H,H) = 7.3, Acenap 7-H), 7.99 (1 H, d, ^3^*J* (H,H) = 7.6, Acenap 3-H), 7.79–7.65 (3 H, m, Acenap 8-H, Te*Ph* 12,16-H), 7.56–7.50 (1 H, m, Te*Ph* 14-H), 7.50–7.41 (2 H, m, Te*Ph* 13,15-H), 7.39–7.23 (3 H, m, Se*Ph* 19-21-H), 7.16–7.05 (2 H, m, Se*Ph* 18,22-H), 3.78–3.58 (4 H, m, 2 × C*H*_2_); ^13^C-NMR [67.9 MHz, (CD_3_)_2_CO, 25 °C, TMS]: *δ* = 153.8(q), 152.4(q), 140.6(s), 137.1(q), 134.6(s), 132.4(q), 132.1(s), 132.0(s), 130.6(q), 130.0(s), 129.9(s), 128.9(q), 128.7(s), 128.4(s), 122.4(q), 121.9(q), 30.3(s, *C*H_2_), 30.0(s, *C*H_2_); ^77^Se-NMR [51.5 MHz, (CD_3_)_2_CO, 25 °C, PhSeSePh]: *δ* = 322.6(s); ^125^Te-NMR (81.2 MHz, (CD_3_)_2_CO, 25 °C, PhTeTePh): *δ* = 544.1(s); ^19^F-NMR [376.5 MHz, (CD_3_)_2_CO, 25 °C, CCl_3_F]: *δ* = −151.6(br s, ^10^BF_4_^−^), −151.7(br s, ^11^BF_4_^−^); IR (KBr disk): *v*_max_ cm^−1^: 2859w, 2681w, 2366w, 2199w, 1692w, 1872w, 1814w, 1734w, 1720w, 1703w, 1655vs, 1641vs, 1589s, 1571s, 1553w, 1542w, 1505w, 1474s, 1435s, 1419s, 1352w, 1327s, 1296w, 1276w, 1257w, 1229w, 1183w, 1053vs, 1019vs, 996vs, 903w, 845s, 813s, 764w, 735vs, 687s, 665w, 637w, 614w, 598w, 572w, 519w, 489w, 471w, 452s, 394w, 337w; MS (ES^+^): *m/z* (%): 1134.24 (50) [M^+^−BF_4_^−^]; elemental analysis calcd (%) for C_48_H_36_AgBF_4_Se_2_Te_2_: C 47.2, H 3.0; Found: C 47.1, H 3.1.

*[AgCF_3_SO_3_{Acenap(SePh)(SPh)}_3_]* (**4**). To a solution of AgCF_3_SO_3_ (0.13 g, 0.51 mmol) in dichloromethane (20 mL) was added [Acenap(SePh)(SPh)] (0.22 g, 0.51 mmol) in one batch at −30 °C. The reaction mixture was stirred at this temperature for 3 h and then at room temperature for a further 12 h. The solvent was removed *in vacuo* to afford the product as a white crystalline solid. An analytically pure sample was obtained by recrystallization from diffusion of hexane into a saturated dichloromethane solution of the product (0.26 g, 67%); m.p. 72–74 °C (decomp); ^1^H-NMR [270 MHz, CDCl_3_, 25 °C, TMS]: *δ* = 7.79 (2 H, d, ^3^*J* (H,H) = 7.3, 2 x Acenap 4-H), 7.57–7.47 (4 H, 2 × Se*Ph*12,16-H), 7.38–7.28 (2 H, m, 2 × Se*Ph* 14-H), 7.28–7.18 (4 H, m, 2 × Se*Ph* 13,15-H), 7.13–7.00 (8 H, m, 2 × Acenap 3-H, 2 × S*Ph* 19-21-H), 7.00–6.94 (4 H, m, 2 × S*Ph* 18,22-H), 6.94–6.86 (4 H, m, 2 × Acenap 7,8-H), 3.25–3.11 (8 H, m, 2 × C*H*_2_C*H*_2_); ^13^C-NMR [67.9 MHz, CDCl_3_, 25 °C, TMS]: *δ* = 151.5(q), 148.3(q), 142.0(q), 141.9(s), 137.5(q), 136.8(s), 133.1(s), 131.8(q), 130.8(s), 130.5(s), 130.1(s), 128.1(q), 127.7(s), 127.6(s), 123.0(q), 121.6(s), 121.5(s), 118.5(q), 30.9(s, 2 x *C*H_2_), 30.3(s, 2 × *C*H_2_); ^77^Se-NMR [51.5 MHz, CDCl_3_, 25 °C, PhSeSePh]: *δ* = 369.9 (s); ^19^F-NMR [376.5 MHz, CDCl_3_, 25 °C, CCl_3_F]: *δ* = -77.9(s); IR (KBr disk): *v*_max_ cm^−1^: 2923w, 2833w, 2344w, 1955w, 1873w, 1803w, 1720w, 1656w, 1597s, 1575s, 1476s, 1439s, 1412s, 1358w, 1330s, 1289vs, 1232vs, 1162vs, 1112s, 1069s, 1023vs, 841s, 739vs, 687vs, 633vs, 572w, 514s, 463w, 401w, 349w, 311w; MS (ES^+^): *m/z* (%): 524.62 (20) [C_24_H_18_S_2_Ag^+^], 941.74 (100) [(C_24_H_18_S_2_)_2_Ag^+^]; elemental analysis calcd (%) for C_73_H_54_AgF_3_O_3_S_4_Se_3_: C 58.0, H 3.6; Found: C 57.9, H 3.5.

*[AgCF_3_SO_3_{Acenap(TePh)(SPh)}_3_]* (**5**). Complex **5** was obtained following the method described previously for **4** but with AgCF_3_SO_3_ (0.14 g, 0.54 mmol), [Acenap(TePh)(SPh)] (0.25 g, 0.54 mmol). An analytically pure sample was obtained by recrystallization from diffusion of hexane into a saturated dichloromethane/methanol solution of the product (0.21 g, 70%); m.p. 200–202 °C(decomp); ^1^H-NMR [270 MHz, CD_3_CN, 25 °C, TMS]: *δ* = 7.91 (3 H, d, ^3^*J* (H,H) = 7.2, 3 × Acenap 4-H), 7.83–7.72 (6 H, m, 3 × Te*Ph* 12,16-H), 7.61–7.50 (3 H, m, 3 × Te*Ph* 14-H), 7.44 (3 H, d, ^3^*J* (H,H) = 7.2, 3 × Acenap 7-H), 7.42–7.32 (6 H, m, 3 × Te*Ph* 13,15-H), 7.32–7.23 (6 H, m, 3 × S*Ph* 19,21-H), 7.23–7.15 (3 H, m, 3 × S*Ph* 20-H), 7.11–7.02 (6 H, m, 3 × Acenap 3,8-H), 7.02–6.94 (6 H, m, 3 × S*Ph* 18,22-H), 3.45–3.31 (12 H, m, 6 × C*H*_2_); ^13^C-NMR [67.9 MHz, CD_3_CN, 25 °C, TMS]: *δ* = 151.8(q), 148.7(s, *C*F_3_), 141.0(s), 140.3(q), 140.2(s), 137.1(q), 135.5(q), 135.4(s), 130.5(s), 130.3(q), 130.1(s), 129.8(q), 129.6(s), 126.5(s), 126.2(s), 121.8(s), 121.2(s), 119.6(q), 100.0(q), 30.2(s, *C*H_2_), 29.6(s, *C*H_2_); ^125^Te-NMR [81.2 MHz, CD_3_CN, 25 °C, PhTeTePh]: *δ* = 552.3(s); ^19^F-NMR [376.5 MHz, CD_3_CN, 25 °C, CCl_3_F]: *δ* =-79.8(s); IR (KBr disk): *v*_max_ cm^−1^: 2921w, 2834w, 2720w, 2643w, 2521w, 2372w, 2345w, 2242w, 2154w, 1946s, 1923s, 1895s, 1682w, 1667w, 1647w, 1633w, 1595w, 1573w, 1583w, 1519w, 1475s, 1435s, 1409w, 1334s, 1293vs, 1229vs, 1165vs, 1111s, 1064w, 1023vs, 997s, 912w, 843s, 817w, 784w, 737vs, 689s, 634vs, 574s, 515s, 489w, 454w, 402w, 353w, 320w; MS (ES^+^): *m/z* (%):574.45 (100) [M^+^−CF_3_SO_3_^−^]; elemental analysis calcd (%) for C_25_H_19_AgF_3_O_3_S_2_Te: C 41.5, H 2.5; found: C 41.5, H 2.4.

*[AgCF_3_SO_3_{Acenap(TePh)(SePh)}]_n_* (**6**). Complex **6** was obtained following the method described previously for **4** but with AgCF_3_SO_3_ (0.09 g, 0.35 mmol), [Acenap(TePh)(SePh)] (0.18 g, 0.35 mmol). An analytically pure sample was obtained by recrystallization from diffusion of hexane into a saturated dichloromethane solution of the product (0.18 g, 67%); mp 125–127 °C (decomp); ^1^H-NMR [270 MHz, CDCl_3_, 25 °C, TMS]: *δ* = 7.97 (1 H, d, ^3^*J* (H,H) = 7.2, Acenap 4-H), 7.82–7.73 (2 H, m, Te*Ph* 12,16-H), 7.45–7.36 (1 H, m, Te*Ph* 14-H), 7.28–7.20 (2 H, m, Te*Ph* 13,15-H), 7.17–7.08 (4 H, m, Acenap 3-H, Se*Ph* 19-21-H), 7.08–7.01 (3 H, m, Acenap 7-H, Se*Ph* 18,22-H), 6.93 (1 H, d, ^3^*J* (H,H) = 7.5, Acenap 8-H), 3.30–3.20 (4 H, m, 2 × C*H*_2_); ^13^C-NMR [67.9 MHz, CDCl_3_, 25 °C, TMS]: *δ* = 151.5(q), 149.0(s, *C*F_3_), 142.9(s), 142.0(q), 140.7(s), 137.1(s), 135.3(q), 135.1(q), 131.0(s), 130.6(s), 130.5(s), 129.2(q), 128.8(s), 128.0(s), 122.2(s), 121.6(s), 120.5(q), 120.3(q), 117.2(q), 108.7(q), 30.8(s, *C*H_2_), 30.2(s, *C*H_2_); ^77^Se-NMR (51.5 MHz, CDCl_3_, 25 °C, PhSeSePh): *δ* = 321.6(s); ^125^Te-NMR [81.2 MHz, CDCl_3_, 25 °C, PhTeTePh]: *δ* = 537.2(s); ^19^F-NMR [376.5 MHz, CDCl_3_, 25 °C, CCl_3_F]: *δ* = −78.1(s); IR (KBr disk): *v*_max_ cm^−1^: 2926w, 2831w, 2373w, 2345w, 2244w, 1972w, 1873w, 1812w, 1739w, 1637w, 1595s, 1572s, 1474s, 1436s, 1420s, 1330s, 1283vs, 1224vs, 1171vs, 1104w, 1062w, 1021vs, 996s, 842s, 814w, 777w, 735vs, 688s, 665w, 633vs, 573w, 515s, 453w, 351w, 319w; MS (ES^+^): *m/z* (%): 620.30 (100) [M^+^−CF_3_SO_3_^−^]; elemental analysis calcd (%) for C_25_H_18_F_3_O_3_STeSe: C 39.0, H 2.4; Found: C 39.1, H 2.3.

### 3.2. Crystal Structure Analyses

X-ray crystal structures for **1**–**6**, **2a** were collected at −180(1) °C by using a Rigaku MM007 High brilliance RA generator (Mo Kα radiation, confocal optic) and Mercury CCD system. At least a full hemisphere of data was collected using ω scans. Data were collected for **3a** at −148(1) °C using a Rigaku MM007 High brilliance RA generator (Mo Kα radiation, confocal optic) and Saturn CCD system. At least a full hemisphere of data was collected using ω scans. Intensities were corrected for Lorentz, polarisation and absorption. The data for the complexes was collected and processed using CrystalClear (Rigaku) [75]. The structures were solved by Patterson or direct methods [76] and expanded using Fourier techniques [77]. Non-hydrogen atoms were refined anisotropically, and hydrogen atoms were refined using a riding model. All calculations were performed using the CrystalStructure [78] and SHELXL-97 [79]. These X-ray data can be obtained free of charge via www.ccdc.cam.ac.uk/conts/retrieving.html or from the Cambridge Crystallographic Data centre, 12 Union Road, Cambridge CB2 1EZ, UK; fax (+44) 1223-336-033; e-mail: deposit@ccdc.cam.ac.uk using CCDC Nos: 873010, 873011, 873015-873019 and 873022.

## 4. Conclusions

Six silver(I) coordination complexes **1**–**6** have been prepared and structurally characterised, based on the self-assembly of mixed chalcogen-donor acenaphthenes [Acenap(EPh)(E'Ph)] (Acenap = acenaphthene-5,6-diyl; EE' = SeS, TeS, TeSe) **L1**–**L3** [49,50,51] with silver(I) salts (AgBF_4_, AgOTf). Modification of the chalcogen donor functionalities within the ligand shell, the coordinating ability of the respective counter-anion and the solvents used during the recrystallisation process all have a significant impact on the structural architecture of the final complex, generating two- and three-coordinate monomeric, mononuclear silver(I) complexes (**1**–**5**, **2a**, **3a**) and a 1D polymeric chain incorporating a left-handed [(-Ag-Te-C-C-C-Se-Ag-)_n_] helix (**6**). Table 6 compares the structural characteristics of **1**–**6**, **2a**, **3a** based on the silver(I) coordination geometry, the ligation modes of **L1**–**L3** and the nature of the donor atoms. In the seven monomeric complexes **1**–**5**, **2a**, **3a**, the acenaphthene ligand binds to the central silver atom via classical monodentate coordination and exclusively via the least electronegative chalcogen congener. In contrast, the tellurium-selenium **L3** ligand in the polymeric chain **6** coordinates simultaneously via both chalcogen atoms in a bis-monodentate μ_2_-η^2^-bridging ligation mode. In all complexes silver adopts either a tetrahedral or trigonal planar geometry in the primary coordination sphere. In addition, the BF_4_^−^ and CF_3_SO_3_^−^ counteranions in monomeric complexes **1**–**5**, **2a**, **3a** were shown to be strictly non-coordinating, whilst the triflate anion in **6** binds to the central silver atom via a single O atom to complete the trigonal planar geometry.

## Supplementary Materials

Supplementary materials can be accessed at: http://www.mdpi.com/1420-3049/17/11/13307/s1.
