# Supplementary material for: Investigating Silver Coordination to Mixed Chalcogen Ligands
Source: Molecules. 2012 Nov 8;17(11):13307–29. doi: 10.3390/molecules171113307 (PMC6269030; doi:10.3390/molecules171113307)
Supplement: Supplementary file 1 [file molecules-17-13307-s001.pdf]

Supporting Information

Investigating Silver Coordination to Mixed Chalcogen Ligands

Fergus R. Knight, Rebecca A. M. Randall, Lucy Wakefield, Alexandra M. Z. Slawin and J. Derek Woollins*

School of Chemistry, University of St Andrews, St Andrews, Fife, KY16 9ST, UK;
E-mail: jdw3@st-and.ac.uk.

Figure S1. Monomeric sandwich complexes **2** and **3** showing the bent metallocene motif found at the center of each complex, formed from two η^6-S(phenyl)···Ag interactions (H atoms and solvent molecules omitted for clarity).

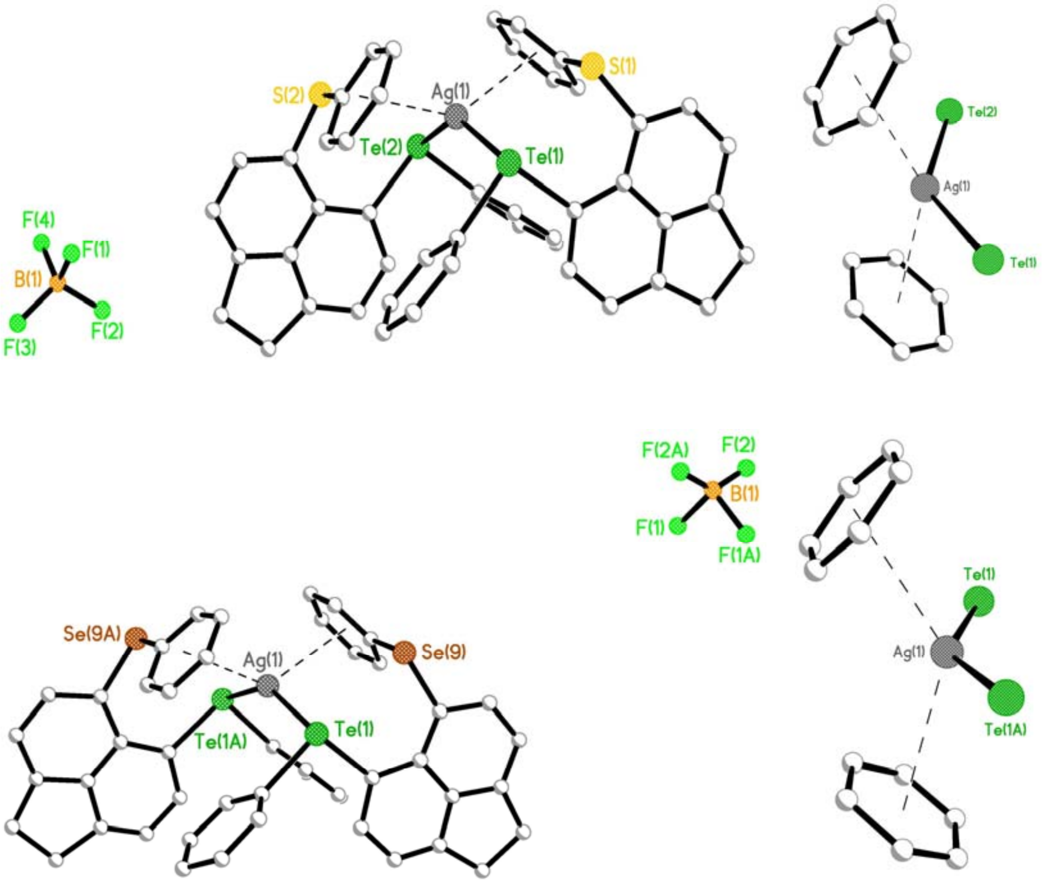

Figure S2. Complexes **2** (left) and **3** (right) viewed down the y-axis; BF_4^- counter-anions stack in channels between the acenaphthene fragments (H atoms and solvent molecules omitted for clarity).

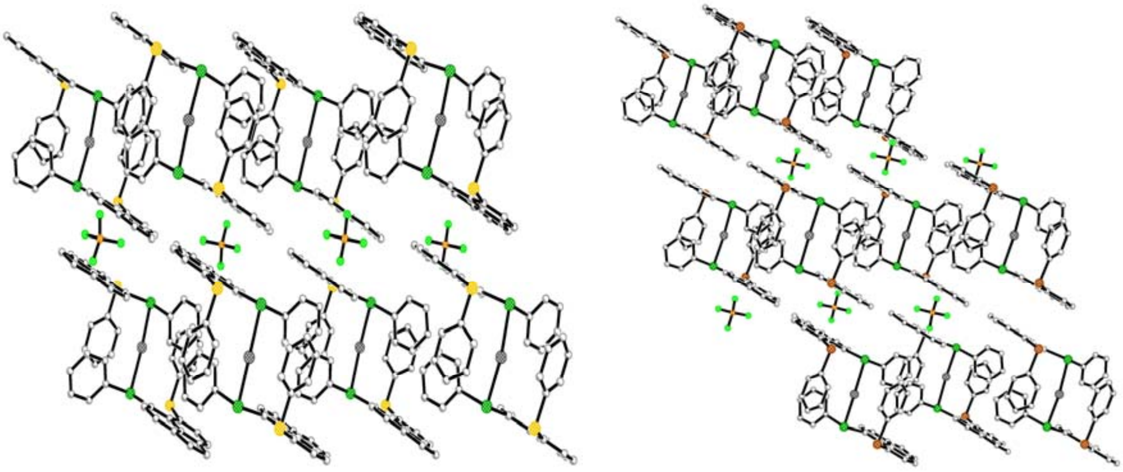

Figure S3. The three coordinate, monomeric, mononuclear silver(I) complex **5**, isomorphous with complex **4**, with disordered triflate molecule (H atoms omitted for clarity).

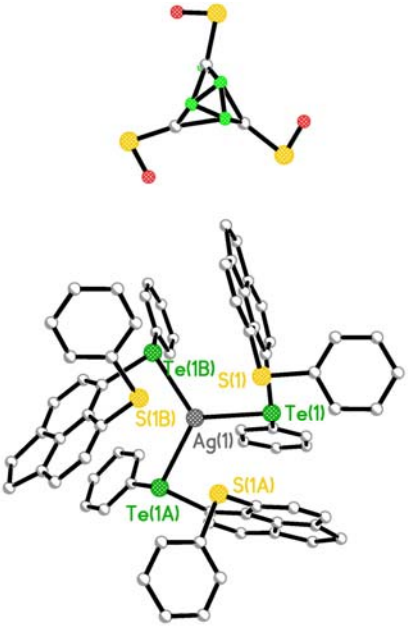

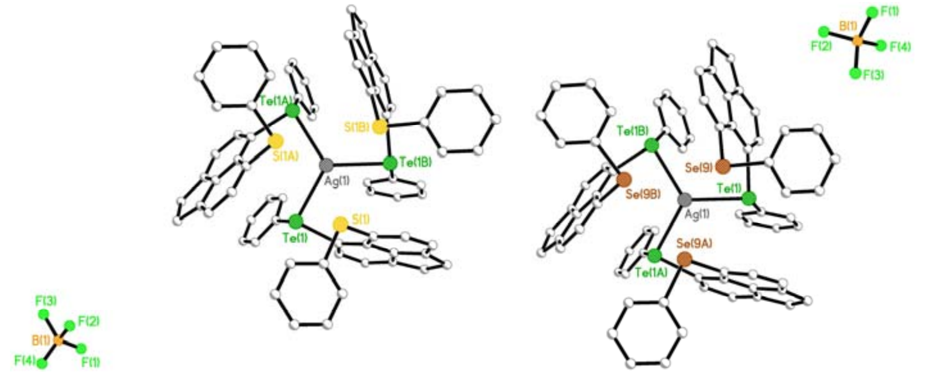

Figure S4. The three coordinate, mononuclear silver(I) complexes **2a** and **3a** adopting similar structural motifs to complexes **4** and **5** (H atoms omitted for clarity).

Table S1. Intermolecular **X-Y···Z** interactions: Distances [Å] and angles [°].

	X-Y···Z	X-Y	Y···Z	X···Z	X-Y···Z		X-Y···Z	X-Y	Y···Z	X···Z	X-Y···Z
**1**	C8-H8···cg(49-54)	0.95	2.79	3.5936	143	**3**	C8-H8···cg(19-24)	0.95	2.85	3.6815	147
	C38-H38···cg(19-24)	0.95	2.79	3.5723	140	**4**	C3-H3···cg(19-24)	0.95	2.62	3.4771	150
	C55-H55B···cg(5-10)	0.95	2.94	3.8932	161		C8-H8···cg(19-24)	0.95	2.91	3.7273	145
	C56-Cl4···cg(35-40)	1.711	3.9123	4.7095	107	**5**	C3-H3···cg(19-24)	0.95	2.68	3.5428	151
	C18-H18···F3	0.95	2.54	3.1247	120		C8-H8···cg(19-24)	0.95	2.9	3.7085	144
	C20-H20···F4	0.95	2.49	3.3907	159	**6**	C12-H12B···cg(5-10)	0.99	2.92	3.6505	131
	C48-H48···F1	0.95	2.54	3.3154	139		C14-H14···cg(19-24)	0.95	2.81	3.4992	130
	C50-H50···Cl4	0.95	2.79	3.4807	130		C24-H24···cg(5-10)	0.95	2.95	3.7376	141
	C53-H53···F4	0.95	2.53	3.3239	142		C8-H8···O1	0.95	2.56	3.398	148
	C54-H54···F3	0.95	2.46	3.3464	154		C11-H11A···O1	0.99	2.40	3.3578	162
	C55-H55A···F1	0.95	2.33	3.2327	151		C23-H23···O2	0.95	2.57	3.3564	140
	C56-H56B···F2	0.95	2.32	3.3076	172	**2a**	C3-H3···cg(19-24)	0.95	2.61	3.4871	154
	C56-H56B···F4	0.95	2.52	3.2584	132		C8-H8···cg(19-24)	0.95	2.79	3.5854	141
**2**	C8-H8···cg(49-54)	0.95	2.76	3.6071	149	**3a**	C3-H3···cg(19-24)	0.95	2.56	3.4467	156
	C38-H38···cg(19-24)	0.95	2.73	3.5752	148		C8-H8···cg(19-24)	0.95	2.71	3.5086	142
	C56-H56A···cg(5-10)	0.95	2.95	3.9253	171						
	C20-H20···F1	0.95	2.45	3.388	156						
	C55-H55A···F2	0.99	2.31	3.2902	173						
	C56-H56B···F3	0.99	2.39	3.2617	146						

Table S2. Selected silver coordination interatomic distances [Å] and angles [°] for **2a**, **3a**.

**Compound**	**2a**	**3a**
Ligand; *peri*-atoms	**L4**; TeS	**L5**; TeSe
Peri-region-distances		
E···E\`	3.1653(18)	3.252(4)
Σr_{vdW} - E···E\`[a]; % Σr_{vdW} [a]	0.695; 82	0.708; 82
Peri-region bond angles		
E(1)-C(1)-C(10)	122.5(5)	123(2)
C(1)-C(10)-C(9)	129.6(7)	131(4)
E\`(1)-C(9)-C(10)	122.3(6)	123(3)
Σ of bay angles	374.4(14)	377(7)
Splay angle[b]	14.4	17
Out-of-plane displacement		
E	0.481(1)	0.482(1)
E\`	−0.101(1)	−0.072(1)
C:(6)-(5)-(10)-(1)	177.28(1)	177.78(1)
C:(4)-(5)-(10)-(9)	176.37(1)	176.47(1)

Table S3. Crystallographic data for **2a**, **3a**.

	**2a**	**3a**
Empirical Formula	$C_{72}H_{54}AgBF_4S_3Te_3·¼H_2O$	$C_{72}H_{54}AgBF_4Se_3Te_3$
Formula Weight	1597.37	1733.57
Temperature (°C)	−180(1)	−148(1)
Crystal Color, Habit	yellow, prism	colorless, platelet
Crystal Dimensions (mm^3)	0.120 × 0.120 × 0.120	0.120 × 0.120 × 0.030
Crystal System	trigonal	trigonal
Lattice Parameters	a = 18.319(4) Å	a = 18.382(4) Å
	-	-
	c = 33.873(9) Å	c = 33.540(7) Å
	-	-
	-	-
	-	-
Volume (Å^3)	V = 9844(5)	V = 9815(3)
Space Group	R-3	R-3
Z value	6	6
Dcalc (g/cm^3)	1.617	1.760
F000	4683	4992
(MoKα) (cm^{-1})	17.618	33.400
No. of Reflections Measured	21078	27110
Rint	0.0530	0.1186
Min and Max Transmissions	0.579–0.809	0.638–0.905
Observed Reflection (No. Variables)	3996(285)	4426(283)
Reflection/Parameter Ratio	14.02	15.64
Residuals: R_1 ($I > 2.00\sigma(I)$)	0.0457	0.1195
Residuals: R (All reflections)	0.0550	0.1457
Residuals: wR_2 (All reflections)	0.1453	0.2882
Goodness of Fit Indicator	1.095	1.421
Maximum peak in Final Diff. Map	1.12 e^-/Å^3	2.07 e^-/Å^3
Minimum peak in Final Diff. Map	−0.71 e^-/Å^3	−2.21 e^-/Å^3
